# Functional Adhesives for a Restorative Future

**DOI:** 10.3390/jfb17070329

**Published:** 2026-07-06

**Authors:** Andreas Katsonis, Monica Silvia Tatarciuc, Anca Mihaela Vitalariu, Roxana Ionela Vasluianu, Jamal Al-Ashkar, Catalina Holban Cioloca, Andrea-Simoni Katsoni, Panagiotis Perperidis, Irina Gradinaru, Adina Oana Armencia, Ovidiu Stamatin

**Affiliations:** 1 Grigore T. Popa University of Medicine and Pharmacy, 700115 Iasi, Romania; md-eng-10374@students.umfiasi.ro (A.K.); tatarciucm@yahoo.com (M.S.T.); anca.vitalariu@umfiasi.ro (A.M.V.); jamal.al-ashkar@d.umfiasi.ro (J.A.-A.); catalinaholban2906@gmail.com (C.H.C.); irina.gradinaru@umfiasi.ro (I.G.); adina.armencia@umfiasi.ro (A.O.A.); ovidiu.stamatin@umfiasi.ro (O.S.); 2 Oral Diagnosis and Radiology, National and Kapodistrian University of Athens, 11527 Athens, Greece; skatsoni@uoa.gr; 3 Private Clinic, 16674 Athens, Greece; perperidis2001@gmail.com

**Keywords:** adhesive cementation, resin cements, lithium disilicate, zirconia, hybrid ceramics, dentin bonding, enamel etching, universal adhesives

## Abstract

Cementation of indirect restorations has undergone a profound change over the past century, evolving from a philosophy of purely mechanical cementation to sophisticated biomaterials science based on adhesion. The aim of this narrative review is to provide essential context for understanding contemporary clinical decision-making. A key aspect is the structural and compositional characteristics of enamel and dentin as bonding substrates, underlining why enamel remains the gold standard, while dentin continues to present significant challenges. The synthesis is based on evidence of adhesive strategies for the four main classes of contemporary restorative materials, such as glass-ceramics (lithium disilicate and leucite), polycrystalline zirconia, resin-matrix ceramics (hybrid ceramics), and indirect composites. Each material was detailed in terms of surface penetration (e.g., hydrofluoric acid etching and air abrasion), chemical coupling (e.g., silanization and MDP-based primers), and appropriate resin cement selection (light-cured, dual-cured, or self-adhesive). A step-by-step clinical protocol is synthesized based on current evidence, integrating critical techniques such as immediate dentin sealing and optimized polymerization. This review concludes that while adhesive resin cements provide predictable long-term results, significant challenges remain, guiding future clinical research and innovation.

## 1. Introduction

The history of cementation in indirect restorative dentistry reflects a significant shift in how clinicians and scientists view the tooth–restoration interface. What originated as a purely mechanical process, where cements served merely to occupy space between two rigid, retentively prepared surfaces, has transformed into a biomaterial-driven field focused on adhesion, interface durability, and minimally invasive principles. This transition from passive luting to active bonding provides the essential historical and scientific context for understanding the rationale behind contemporary cementation procedures.

For most of the 20th century, the success of a crown or bridge depended almost entirely on the geometry of the tooth preparation. The luting agent was a passive filler, and retention was a function of taper, height, and surface area. However, the advent of acid etching, the development of dentin-bonding hybrid layers, and the introduction of high-performance ceramic and resin-based restorative materials have rendered this mechanical philosophy obsolete. Today, cementation is a highly specialized, substrate-specific, and material-specific process. It is no longer a single technique, but a range of tailored strategies designed to create a durable, biomimetic interface that can reinforce brittle restorations, preserve tooth structure, and improve long-term survival.

From a functional materials perspective, modern luting agents are no longer passive space-fillers but active components that must exhibit a complex combination of properties, such as adequate mechanical strength, appropriate modulus of elasticity, hydrolytic stability, chemical adhesion to dissimilar substrates, biocompatibility, and bioactive functionality [1,2]. The interface they create functions as a multi-layered structure that must withstand cyclic mechanical loading, thermal fluctuations, and enzymatic degradation over decades of service.

This narrative review has three primary objectives, which are justified by the current state of clinical and scientific evidence. Despite the availability of modern adhesives, many clinical practices are still influenced by habits formed during the “mechanical era.” A clear understanding of why traditional cements (e.g., zinc phosphate and glass ionomer) fail where modern resin cements succeed is necessary to justify the adoption of more complex, technique-sensitive adhesive protocols [2,3]. We therefore hypothesize that the evolution from mechanical to adhesive cementation is directly correlated with improved clinical outcomes for tooth-supported prostheses, but only when substrate-specific principles are followed.

The market is flooded with diverse restorative materials (e.g., lithium disilicate, zirconia, and hybrid ceramics) and a bewildering array of adhesives and cements. A major source of clinical failure is the mismatch between a restorative material’s surface chemistry and the chosen adhesive protocol (e.g., attempting to etch zirconia with hydrofluoric acid). This review tests the hypothesis that each class of restorative material requires a unique, non-interchangeable combination of micromechanical (e.g., air abrasion, acid etching) and chemical (e.g., silane, MDP) surface pretreatment to achieve a durable bond.

Despite significant advances, problems such as durable dentin bonding, reliable zirconia adhesion, and complete polymerization under thick restorations remain unresolved. By systematically reviewing the limitations of current materials and techniques, this review aims to justify and direct future research toward bioactive, self-healing, and digitally integrated cementation systems [4,5].

## 2. Methodology

This narrative review was conducted to provide a comprehensive overview of functional materials used for adhesive cementation in indirect restorative dentistry. A systematic literature search was performed in PubMed, Scopus, and Web of Science databases for articles published between January 2015 and March 2026. The following search terms were used in various combinations: “dental cement,” “resin cement,” “adhesive cementation,” “cementing agent,” “bond strength,” “zirconium bond,” “lithium disilicate,” “universal adhesive,” “10-MDP,” “dentin bond,” and “bioactive cement.” Inclusion criteria included peer-reviewed articles in English, in vitro studies reporting mechanical or physicochemical properties, clinical trials, systematic reviews, and meta-analyses. Reference lists of included articles were hand-searched to identify additional relevant publications. Given the narrative nature of this analysis, a quantitative meta-analysis was not performed. To address potential selection bias and heterogeneity, where there was conflicting evidence, we prioritized results from systematic reviews and meta-analyses. When these were not available, we reported a range of results from high-quality in vitro studies. A PRISMA flow chart of the search and selection process is shown in Supplementary Materials File S1.

## 3. The Historical Evolution of Cementation: From Mechanical Luting to Adhesive Dentistry

To appreciate the sophistication of contemporary cementation, one must first understand the limitations of the past. This evolution is best understood as three distinct eras, summarized in Table 1.

### 3.1. The Old Luting Era: A Mechanical Philosophy

For most of the 20th century, the cementation process was predominantly understood as a mechanical phenomenon. The cementing agent was merely a passive filling material, and the long-term viability of the prosthetic restoration depended almost entirely on the geometry of the preparation, such as taper, height, and surface area [2,3] (Table 2).

The old era was defined by the limitations of a purely mechanical philosophy, creating the need for a new generation of cementing agents capable of bonding and strengthening [6,12,13,14,15,16,17,18,19].

### 3.2. The Transitory Era: The Rise of Resin Cements

The introduction of resin cements in the 1980s marked a pivotal shift. Formulated with Bis-GMA and other dimethacrylates, they offered superior mechanical strength (flexural strength 80–150 MPa), low solubility (<0.05% after 24 h), and the potential for micromechanical bonding. This allowed for more conservative preparations [3,20].

A critical development was the dual-cure resin cement, designed to polymerize in areas where light penetration is poor (e.g., under thick or opaque crowns). However, research consistently shows that light activation remains critical for optimal conversion. Insufficient light exposure compromises the degree of conversion (reducing from >70% with adequate light to <50% without) and long-term stability [8,21,22]. This era laid the scientific foundation for modern adhesive strategies.

### 3.3. The Modern Adhesive Era: The Engineering of an Interface

The evolution to contemporary bonding represents a fundamental shift from mechanical retention to the design of a resilient, biomimetic interface. Buonocore’s groundbreaking discovery in 1955 that etching with phosphoric acid creates a micro-retentive surface in enamel marked the beginning of this period. People around the world agree that this is when adhesive dentistry began [10,23].

Nakabayashi’s hybrid layer concept was a major step forward for dentin. It is the micromechanical interlocking zone that forms when resin infiltrates demineralized dentin. Early etch-and-rinse systems were susceptible to technical problems and were likely to leak at the nanoscale and degrade in water [7,14].

Development continued through several generations of adhesives, culminating in universal adhesives. Functional monomers such as 10-MDP (10-methacryloyloxydecyl dihydrogen phosphate) are a major reason why they work so well. They produce stable MDP-Ca salts with hydroxyapatite that do not decompose in water [5,11]. The bond strength of 10-MDP-based systems is typically between 20 and 40 MPa for dentin and between 15 and 30 MPa for zirconia. This represents a significant improvement over the previous generation [24,25].

**Figure 1:**
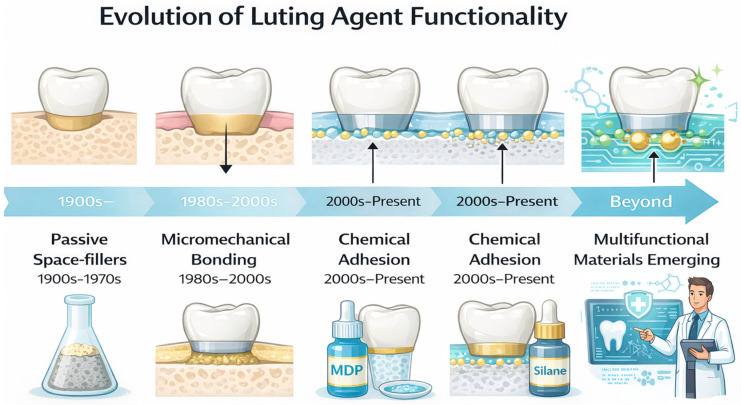
Evolution of luting agent functionality. Timeline from passive space-fillers (1900s–1970s) to micromechanical bonding (1980s–2000s) to chemical adhesion (2000s–present) to emerging multifunctional materials (bioactive, self-healing, digitally integrated).

The most important change in thinking during this period is the realization that adhesion is substrate-dependent, as shown in Figure 1.

## 4. The Biological Substrates

The success of any adhesive cementation procedure depends on a thorough understanding of the dental substrate. Enamel and dentin are fundamentally different in terms of structure, composition, and adhesion.

### 4.1. Enamel as a Substrate

Enamel remains the most predictable and durable substrate for adhesive dentistry due to its highly mineralized, acellular structure. Histologically, it is composed of approximately 92–95% vol. hydroxyapatite, providing a stable surface for resin infiltration. The foundation of modern cementation is the Buonocore acid etching technique, which produces a microporous surface that facilitates durable hybridization, with reported bond strengths of 25–40 MPa [4,26].

However, adhesion to aprismatic enamel, commonly found on unpolished surfaces or in cervical regions, is less predictable, with bond strengths reduced by up to 50%. Selective etching of enamel prior to application of self-etching adhesive has been repeatedly shown to overcome this limitation and improve fracture resistance [27]. Regarding surface degradation, although erosion significantly impairs dentin adhesion, bond strength to eroded enamel is not consistently reduced (typically within 10–20% of healthy enamel), although prolonged erosion may compromise dental resin formation [28]. Ultimately, enamel preservation is a determinant of restoration longevity, and maximizing enamel contact through selective etching remains an essential strategy [29].

### 4.2. Dentin as a Substrate

Dentin is a significantly more difficult material to bond to, as it is composed of approximately 45% minerals, 33% organic matrix (mostly type I collagen) and 22% water. It also has a dynamic, hydrated tubular structure. This environment makes resin infiltration difficult and makes dentin adhesion less predictable. Bond strength is typically between 15 and 30 MPa, while bond strength for enamel is between 25 and 40 MPa [4,26,30].

The complication is further compounded under pathological circumstances. Erosion reduces dentin bond strength by 30–60%, resulting in a demineralized, collagen-rich superficial layer that is prone to collapse and inhibits the proper formation of a hybrid layer [28,31]. It is also well known that dentin can degrade over time when exposed to water. After 6 to 12 months of storage in water, bond strength can decrease by 20 to 50% as the collagen fibrils and resin components of the hybrid layer degrade [4,32]. To reduce these weaknesses, the immediate dentin sealing (IDS) method has become an important option. Applying the adhesive immediately after tooth preparation creates a stable hybrid layer before impression taking. This makes the bond last longer, keeping the hybrid layer intact and reducing sensitivity after surgery [33,34,35]. A systematic review confirms that IDS increases bond strength and decreases marginal detachment compared with delayed sealing methods [36]. Regarding clinical implementation, when using IDS cements, the choice of provisional cement is critical; cements containing eugenol should be avoided as they inhibit adhesive polymerization. If a provisional cement containing eugenol was used, the IDS layer should be cleaned with 50 µm aluminum oxide and re-etched before final cementation. For provisional cements without eugenol, light cleaning with pumice is usually sufficient [20].

**Figure 2:**
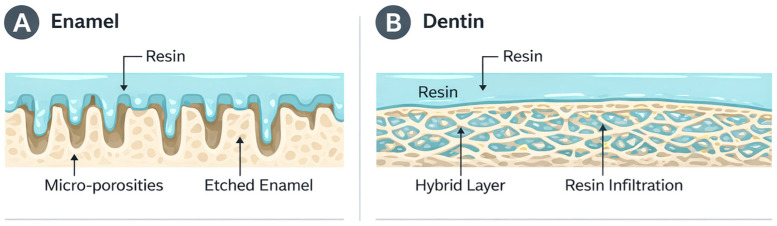
Schematic illustration of the contemporary adhesive interface. (**A**) Enamel: Resin tags penetrating etched enamel prisms. (**B**) Dentin: Hybrid layer with resin infiltration into demineralized collagen network.

Sclerotic dentin presents a unique clinical problem, frequently seen in non-carious cervical lesions. This hyper-mineralized dentin with blocked tubules is very difficult to etch, and the bond strength is 40–60% lower than that of normal dentin. Sclerotic lesions frequently have a hyper-mineralized superficial layer that inhibits sufficient acid penetration and resin infiltration, thus requiring additional surface pretreatment methods [37]. In conclusion, dentin sealing is still the weakest part of adhesive dentistry to achieve predictable, long-term clinical results, careful techniques and methods, such as IDS [38,39] (Figure 2).

## 5. Physicochemical Properties of Functional Luting Agents

The performance of a cementing agent is governed by its physicochemical properties, which determine its handling characteristics, polymerization behavior, mechanical integrity, and long-term stability. The physicochemical properties of functional luting agents, particularly their adhesion to tooth substrates, can be influenced by surface treatments, while the application of preheated resin composites as luting agents offers potential improvements in their handling and mechanical characteristics [40,41,42]. Table 3 summarizes the key properties of contemporary classes of cementing materials (Table 3).

### 5.1. Polymerization Kinetics and Degree of Conversion

The degree of conversion (DC) is an important factor influencing mechanical characteristics, dimensional stability, and biocompatibility. Under ideal circumstances, dual-cure resin cements exhibit DC values between 55 and 75%. However, these values can diminish to below 50% when light activation is inadequate [22,46,62]. This decrease is of clinical importance, as insufficient DC is associated with elevated monomer release, thereby raising concerns regarding pulp irritation and the long-term success of restorations [39,50]. Preheated composites, utilized as luting agents, attain DC values ranging from 60–80% due to enhanced monomer mobility at elevated temperatures (typically 55–68 °C) [47,63]. Furthermore, preheating diminishes film thickness and improves adaptation to the tooth structure, thereby augmenting bond durability [64,65,66]. The existence of oxygen-inhibited layers at the restoration–cement interface can reduce surface DC by 10–20%, thereby compromising marginal integrity [64]. This incompletely polymerized layer not only weakens the adhesive interface but also serves as a preferred site for biofilm accumulation and microleakage, thus highlighting the significance of adequate light exposure through the restorative material [65,66,67].

This vulnerability to inadequate light activation is particularly critical when cementing highly translucent zirconia or lithium disilicate restorations, as the crystal structure of the ceramic can attenuate up to 30–40% of the incident light, effectively reducing the curing efficiency at the cement interface [51,54,68]. In addition, the chemical initiator system in dual-cure cements (tertiary amines associated with benzoyl peroxide) is sensitive to acidic monomers commonly present in self-adhesive formulations, which can lead to premature decomposition and reduced contribution to dark cure when light penetration is compromised [2,37,48]. Although 68 °C is at the upper limit of recommended commercial or research-based preheating, many studies find that preheating to 54–68 °C reduces viscosity, increases flowability, and improves degree of conversion (DC) [69,70,71].

In addition, prolonged heating cycles have been shown to degrade the activity of the camphor quinone photo-initiator, requiring strict adherence to the manufacturer’s specified preheating protocols [72].

### 5.2. Physical Properties

The ability of cement to withstand masticatory forces and distribute stress across the contact is measured by its flexural strength and modulus of elasticity. Compared with conventional cements, adhesive resin cements (100–150 MPa) and heated composites (120–180 MPa) have better mechanical properties [20,35]. However, if the modulus is too high (more than 18 GPa), it can put too much pressure on the contact, which could cause delamination [73]. The best values of modulus are considered to be between 10 and 15 GPa, which are close to the values for dentin (12–18 GPa) and enamel (40–80 GPa), while still allowing for stress absorption [49].

### 5.3. Absorption and Water Solubility

Absorption and water solubility are important factors affecting long-term hydrolytic stability. Resin-based adhesive cements have the lowest water absorption (15–35 µg/mm^3^) and solubility (0.1–1.0 µg/mm^3^). GIC and RMGIC cements, on the other hand, have much higher values [12,49]. Some universal adhesives are hydrophilic, meaning they can absorb more water over time. This could lead to the breakdown of the hybrid layer by hydrolysis [11]. Functional monomers, such as 10-MDP, improve hydrolytic stability by creating stable ionic bonds with hydroxyapatite, which prevents water penetration [74].

## 6. Mechanisms of Bond Degradation and Interface Durability

**Figure 3:**
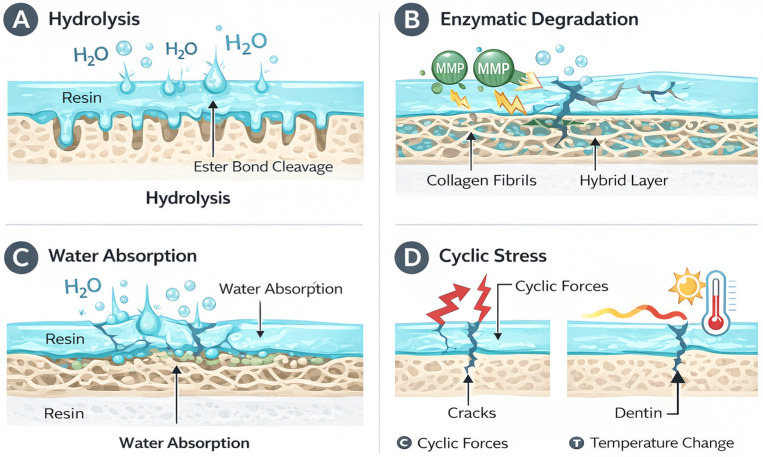
Mechanisms of adhesive interface degradation. (**A**) Hydrolytic cleavage of ester bonds in methacrylate polymers. (**B**) Enzymatic degradation of collagen fibrils by MMPs. (**C**) Water absorption leading to plasticization and swelling. (**D**) Cyclic thermal and mechanical stress causing interfacial crack propagation.

To design useful materials that last longer, it is important to understand how adhesive surfaces degrade over time (Figure 3).

This degradation is a multifactorial process driven by chemical, enzymatic, and mechanical challenges in the dynamic oral environment.

Hydrolytic degradation: Water is the main agent of chemical degradation. Hydrolysis cleaves ester bonds in the methacrylate resin matrix and degrades the exposed collagen fibrils of the hybrid layer. At the same time, water absorption plasticizes the polymer network, decreasing its mechanical properties by 20–40% after 6–12 months of water storage [11,49]. The inclusion of hydrophilic monomers, such as HEMA (hydroxyethyl methacrylate), exacerbates this problem by increasing water sorption.

Enzymatic degradation: Acid etching conditions activate endogenous dentin matrix metalloproteinases (MMPs) and cysteine cathepsins. These enzymes slowly proteolyze the exposed collagen fibrils in the hybrid layer, causing a 30–50% reduction in bond strength over a period of 12–24 months. Application of MMP inhibitors, such as chlorhexidine and benzalkonium chloride, has been shown to effectively attenuate this enzymatic degradation [4].

Thermal and mechanical fatigue: Cyclic thermal variations (5–55 °C, typically 5000 to 10,000 cycles) induce differential expansion between the restorative material, cement, and tooth substrate. This mismatch generates interfacial stresses that weaken the bond by 15–35%. Mechanical fatigue (cyclic loading at 50–200 N for 10^5^–10^6^ cycles) further contributes to interfacial cracking, a critical failure mode in bonded brittle ceramics [75,76].

Galvanic corrosion: Although less relevant for all-ceramic restorations, galvanic currents can form between dissimilar metal restorations or between titanium implants/posts and adjacent structures. These electrochemical couples can accelerate adhesive degradation at the cement–tooth interface [2].

Synergistic degradation: It is worth noting that these degradation mechanisms do not operate in isolation. Hydrolytic swelling can enlarge micro-lacunae, facilitating bacterial infiltration and enzymatic activity. Similarly, mechanical fatigue can expose fresh hydrolytic sites, accelerating chemical degradation [32]. This synergy highlights that complete bond breakage is often the cumulative result of multiple interacting degradation pathways [4,10,32].

Contemporary issue: Current research is increasingly focusing on the complex, site-specific dynamics of degradation in vivo. In contrast to simplified laboratory aging, the oral environment presents a dynamic cocktail of salivary enzymes, cyclical pH fluctuations from dietary acids and bacterial plaque, and a polymicrobial biofilm [2,14]. This biofilm not only produces acids but also secretes bacterial enzymes that can directly degrade adhesive components, a factor often underestimated in vitro. In addition, long-term clinical performance is critically influenced by the quality of the hybrid layer; incomplete resin infiltration leaves a gaping collagen zone vulnerable to both host-derived proteases (MMPs) and bacterial ones. Therefore, contemporary strategies are moving towards “bioactive” adhesives that not only resist degradation but also actively buffer pH, release therapeutic ions (e.g., calcium, phosphate), or incorporate antibacterial monomers to disrupt biofilm formation at the interface, representing the next frontier in improving clinical durability [4,11,32].

## 7. Material-Specific Adhesive Strategies

### 7.1. Glass-Ceramics (Lithium Disilicate & Leucite)

Glass-ceramics require a two-step bonding protocol. The first step consists of etching with hydrofluoric acid (HF) for micromechanical interlocking, followed by the application of silane for chemical coupling [77]. Etching parameters are critical, namely, insufficient etching compromises bond strength, while excessive etching degrades the flexural strength of the ceramic [77]. Resin cements containing MDP monomers demonstrate superior chemical interaction with silanized glass surfaces compared to self-adhesive alternatives [78].

The cementing agent significantly influences the final shade, especially for thin laminates (0.3–0.5 mm), where the cement layer exerts the greatest color impact [79,80]. Trial pastes help to evaluate shade, but do not perfectly reproduce the final appearance of the resin cement. Preheated composite resins offer a viable alternative, demonstrating equivalent or improved color stability while maintaining adequate mechanical properties [81,82].

The mode of polymerization directly affects bond strength, with dual-curing systems providing a more reliable bond to lithium disilicate than single-cured formulations, particularly for thicker or highly translucent restorations with low light transmission [83].

Resin cements and flowable composites improve ceramic performance by infiltrating surface microdefects and transferring occlusal stresses, increasing fracture resistance, which is particularly important for minimally invasive preparations with reduced ceramic thickness [84]. Dual-cure resin cements provide superior marginal sealing, reducing microleakage and postoperative sensitivity compared to conventional cements [85] (Table 4).

### 7.2. Zirconia (Polycrystalline)

Zirconia is a polycrystalline ceramic and has no glass phase and is resistant to hydrofluoric acid etching. Achieving durable adhesion requires both micromechanical retention through surface modification and chemical bonding between the resin cement and the zirconium substrate [90,91].

Zirconia is available in several generations with distinct properties. Conventional 3Y-TZP has high flexural strength (~1200 MPa) but limited translucency and is prone to phase transformation under excessive abrasion pressure (>0.3 MPa) or particle sizes > 110 µm [61,91]. 4Y-TZP has intermediate strength (~1000 MPa), while 5Y-TZP has maximum translucency but at the expense of strength (~700 MPa) and is less prone to phase transformation, though more vulnerable to surface chipping [61,91]. Abrasion of airborne particles with Al_2_O_3_ (50–110 µm) is the gold standard for creating micromechanical retention, increasing surface energy and wetting [92,93]. However, parameters must be adapted for the generation of zirconia: 4Y- and 5Y-TZP need lower pressure (0.2 MPa) and smaller particles (30–50 µm) to prevent surface damage [61,77]. Saliva contamination should be removed before abrasion, since residual organic material compromises bond formation. Micromechanical retention is insufficient.

Resin cements containing the monomer 10-MDP are essential, as the phosphate group builds stable ionic bonds with zirconium oxides [94]. This chemical interaction significantly enhances bond strength and hydrolytic stability [95]. Universal adhesives with MDP are effective, although some studies have shown that MDP-based cements can achieve comparable bonds without an additional adhesive layer [96,97]. Chemical etching solutions with hydrofluoric acid selectively etch the zirconia grain boundaries, achieving bond strengths equal to or exceeding those of air abrasion without the risk of phase transformation [98,99]. Additionally, the application of a silica-based ceramic layer on the inlay surface allows for conventional silanization, expanding adhesive options [2,100,101] (Table 5).

### 7.3. Resin-Matrix Ceramics/Hybrid Ceramics (e.g., PICN)

Resin matrix ceramics, including polymer-infiltrated ceramic network (PICN) materials, possess an organic–inorganic composition that requires a fundamentally different bonding approach compared to glass-ceramics. Hydrofluoric acid etching is not effective due to the limited ceramic phase and the polymer matrix’s resistance to uniform acid dissolution [108,109,110]. Surface treatment is based on particle abrasion in air with 50 µm aluminum oxide, which results in a 40–60% increase in bond strength compared to untreated surfaces by creating a microroughness that facilitates resin infiltration [109]. This mechanical activation is critical for the establishment of durable micromechanical retention. The role of silane is less definite for resin matrix ceramics due to the limited ceramic content, which diminishes the silanol groups available for condensation reactions [109,111,112].

Contemporary universal adhesives containing 10-MDP have emerged as the preferred chemical coupling strategy, achieving bond strengths of 20–35 MPa. MDP has a double affinity for the exposed ceramic phases and can copolymerize with the polymer matrix, thus eliminating the need for a separate silanization step [112,113]. The highest and most predictable bond strengths of adhesive resin cements are achieved in combination with pretreatment and universal adhesive.

Self-adhesive resin cements show significantly lower bond strengths (10–20 MPa) when used without an additional adhesive system because they lack the acidic monomers to condition the resin-rich surface [114,115]. High-viscosity preheated composites have also been proposed by taking advantage of the improved penetration of monomers into the abraded surface [110].

A successful cementation follows a sequential approach, namely air abrasion with 50 µm Al_2_O_3_ for micromechanical retention, a universal adhesive (containing 10-MDP) for chemical bonding, and resin-based adhesive cement or a preheated composite for cementation. Omitting sandblasting or using self-adhesive cements without pretreatment leads to a clinically relevant reduction in bond strength, especially under thermal and mechanical loading [111,116] (Table 6).

### 7.4. Indirect Composite Restorations

The durability of indirect composite restorations relies on a dual-bonding approach to both the resin matrix and exposed filler particles, a concept that deviates significantly from ceramic bonding.

The most proven technique involves abrasion with 50 µm suspended aluminum oxide particles, which removes contaminants and creates a micro-retentive topography, increasing bond strength by 50–100% over untreated surfaces [109]. Lack of sufficient roughness compromises interfacial cohesion.

Upon post-mechanical activation, application of silane or a universal adhesive leads to covalent bonds with the exposed glass fillers [117]. This chemical coupling improves immediate bond strength and provides essential hydrolytic stability against intraoral water absorption and interfacial degradation.

Preheated composites have higher flexural strengths of 120–180 MPa and filler contents of 70–85% by weight compared to conventional resin cements [35,118] and are associated with improved mechanical performance and wear resistance. However, they have 2–3 times greater bacterial adhesion and therefore careful marginal finishing and increased vigilance against biodegradation are required [118]. Material selection should be individualized according to the site of the restoration, aesthetic requirements, and the patient’s caries risk, balancing mechanical benefits with increased biofilm susceptibility [118] (Table 7).

## 8. Discussion

Active functionality beyond passive adhesion is what defines the next generation of cementing agents. Bioactive materials are engineered to interact positively with the biological environment, encouraging remineralization, suppressing bacterial growth, and possibly promoting tissue regeneration [1,119]. To offer clinically useful advice, these emerging technologies need to be clearly differentiated by their commercial availability, translational barriers, and comparative performance against current standards. Among the commercially available choices, ion-releasing cements, such as Activa Bioactive (Pulpdent) and Ceramir (Doxa), release calcium, phosphate, and fluoride ions to inhibit secondary caries. However, their mechanical properties are still inferior to resin-based systems and long-term clinical data are scarce. Bioactivity is evident in calcium silicate cements such as Biodentine, but their low flexural strength (30–50 MPa) limits their use in light-duty fillings [1,120,121].

Several experimental bioactive systems remain in the preclinical stage. Hydroxyapatite nanoparticles incorporated into magnetic glucuronate cements (GICs) improve compressive strength by 20–30% [17,119], while bioactive resin cements aim to combine mechanical strength (80–120 MPa) with sustained ion release (2–5 µg/cm^2^ fluoride/24 h) [122]. However, no truly bioactive resin cement with both high strength (>100 MPa) and sustained ion release is commercially available. Challenges include stable ion release over time without loss of mechanical properties, regulatory issues for bioactivity claims and higher costs of the material. Antibacterial monomers, such as quaternary ammonium methacrylates, provide a dual therapeutic effect by preventing biofilm formation without loss of mechanical properties [122]. However, all data are still laboratory-based, with no available commercial luting cement using this technology. Self-healing materials are based on microcapsules (50–200 µm, 5–15 wt%) filled with TEGDMA that break upon crack formation, polymerizing to seal the defect. Laboratory studies demonstrate a 50–80% recovery of original mechanical properties [123,124,125]. However, no commercially available product exists, and all data remain preclinical due to barriers including capsule–matrix compatibility, healing efficiency under cyclic loading, and lack of regulatory pathways.

While these materials offer bioactive benefits, they cannot match the mechanical performance of modern resin cements and are best for low-stress situations where bioactivity outweighs strength. Surface functionalization technologies provide direct clinical advantages. UV-mediated photofunctionalization (360–400 nm, 5–15 min) increases the surface energy of zirconia by 50–100%, improving bond strength by 20–40% through photocatalytic decomposition of surface hydrocarbons [126,127,128]. This technology is commercially available, easy to implement, and adds minimal clinical time.

Digital workflow integration has been extended by the introduction of preheated composite delivery systems (55–68 °C) to reduce viscosity by 50–80% and improve the fit [129,130], as well as automated mixing to ensure constant proportions and 3D-printed prostheses that need customized cementation protocols for the new materials [131,132,133]. These technologies are commercially available and clinically mature, offering immediate advantages without any fundamental changes in established adhesive principles. Evidence on clinical performance indicates that resin-based adhesive cements remain the gold standard, offering 5-year survival rates of 94–98% for lithium disilicate, 92–96% for zirconia crowns with MDP-containing cements (as compared to 85–90% without cements), and 90–95% for indirect composites [134,135,136,137,138,139,140]. The most common failure modes include delamination (30–50% of failures, mainly at the dentin–cement interface), ceramic/composite fracture, secondary caries (more common with non-adhesive cements), and postoperative sensitivity (more common with etch-and-rinse systems) [2,28,135,141].

Although adhesive cements cost 3–5 times more than conventional alternatives, they become cost-effective when restoration survival exceeds 5–7 years. However, the sensitivity of the technique and the additional clinical time remain barriers to widespread adoption [142,143].

Specific substrate surface management is essential for optimal results. For zirconia, air abrasion remains the gold standard, increasing bond strength by 50–100%, although contamination severely compromises the bond [108,144,145,146,147]. For lithium disilicate, hydrofluoric acid etching combined with silane application provides the most predictable bond through micromechanical interlocking and covalent bonding with siloxane [148,149]. For dentin, IDS preserves the substrate, reduces postoperative sensitivity, and improves marginal integrity. Composite-reinforced flowable IDS further increases bond strength [34,36,150,151,152].

In terms of luting agent selection, resin-based adhesive cements remain the gold standard for nonretentive preparations under high stress due to their better retentiveness and ceramic hardening. Self-adhesive cements provide a clinically comfortable solution but present a higher failure rate when the etching of enamel is not performed and are therefore mostly used in posterior molars with macromechanical retention or in cases where isolation is difficult [153,154]. Preheated composites with high viscosity have better mechanical properties but require the use of additional equipment and demonstrate 2–3 times more bacterial adhesion [119,155,156,157]. Self-adhesive cements present acceptable but suboptimal performance (85–90% survival at 5 years), while preheated composites present a better mechanical profile but an increased risk of biofilm formation.

The dynamics of polymerization also affect the results. The polymerization mode is essential for the polymerization of resin-based luting cements, with light-curing systems exhibiting better wear resistance than dual-curing systems. The COMBO technique offers a simplified approach to cementation, using a high-viscosity bulk-fill composite that achieves adequate polymerization due to its optimal light transmission and reduced thickness [158,159,160,161]. The step-set technique, which includes partial polymerization before final cementation, reduces marginal discrepancies, especially in translucent restorations, while selective adhesive cementation decreases interfacial stress without affecting bond strength [162,163,164]. Strict moisture control is required regardless of the polymerization technique [165].

New considerations arise with digital workflows and CAD/CAM materials, and fiber-reinforced composites require careful selection of surface pretreatment [166,167]. The resin-coating technique is a thin layer of unfilled resin applied to the prepared tooth, which protects the dentin surface and aids cementation for CAD/CAM materials [33].

Novel bioactive formulations with calcium phosphate nanoparticles and quaternary ammonium methacrylates are shifting the cementation interface from a passive mechanical connection to an active protective zone. However, long-term clinical studies are needed as current evidence is largely based on laboratory studies [168,169]. In summary, the current body of evidence supports a shift to customized, substrate-specific adhesive cementation methodologies including mechanical surface preparation, chemical bonding agents and judicious material choices. The previously dominant “one-size-fits-all” approach is giving way to sophisticated strategies considering the specific restorative material, dental substrate, preparation design and functional requirements. Resin adhesive cements remain the gold standard for predictable long-term outcomes. Self-adhesive cements are convenient but at the expense of performance. Bioactive materials have promising adjunctive benefits but are largely preclinical, with the exception of ion-releasing GICs. Digital tools offer immediate clinical benefit and are the most mature emerging technology. Future investigations should focus on long-term clinical studies directly comparing emerging techniques, particularly as bioactive materials and digital workflows are increasingly integrated into routine prosthetic procedures.

## 9. Limitations of This Review

As a narrative review, this work has some limitations, including the potential for selection bias and the lack of systematic, quantitative meta-analysis. The existing literature is highly variable with respect to study design, testing methods, and follow-up periods. Furthermore, many newer materials (e.g., bioactive cements) are supported mainly by in vitro research, and their long-term clinical performance remains uncertain. The bond strength, survival rates, and conversion rates cited are presented without a formal critical assessment of study quality. In vitro bond strength studies often overestimate clinical performance due to idealized conditions and methodological heterogeneity (e.g., storage time, thermal cycling protocols, loading conditions) that limit direct comparisons between studies. We preferred the results of systematic reviews and meta-analyses when they were available; otherwise, data from a single study should be considered preliminary. Finally, adhesive dentistry is a field that is rapidly evolving, and not all recent developments may be fully represented here.

## 10. Future Directions

The future of adhesive dentistry lies in translating mechanistic understanding into durable, clinically tolerant materials. First, degradation-resistant chemicals should be prioritized. Long-term studies should validate MMP inhibitors (e.g., chlorhexidine 2%), while novel monomers (vinylcyclopropanes) and crosslinkers (proanthocyanidins) should target a 40% increase in collagen stability, with ≤10% strength loss after aging. Second, technical insensitivity requires universal primers that provide ≥25 MPa bond strength even in humid conditions, reducing operator variability from 30% to ≤15%. Third, depth-independent polymerization requires redox/thermal initiators that achieve ≥65% conversion through 6 mm of opaque ceramic, with dual-cure cements that offer practical working times and an ultimate modulus ≥8 GPa. Fourth, bioactive multifunctionality should combine ion-releasing fillers (bioglass or calcium phosphates) with antibacterial monomers (quaternary ammonium compounds) to simultaneously combat degradation and infection without sacrificing mechanical integrity. Fifth, self-repairing systems should demonstrate ≥70% bond strength recovery over multiple cycles and limit fatigue-induced losses to ≤20% versus 50% for the control group.

## 11. Conclusions

The progression of cementation techniques in indirect restorative dentistry, from mechanical methods to functional adhesive interfaces, reflects significant developments in materials science and the understanding of tooth–restoration interactions. Modern functional cements are subject to stringent criteria, including sufficient mechanical strength, hydrolysis resistance, chemical adhesion to various substrates, biocompatibility, and, more recently, bioactive properties.

The incorporation of sophisticated functional materials alongside clinically validated protocols presents an opportunity to develop durable biomimetic restorations, thereby preserving tooth tissue and improving patient outcomes over time. Sustained interdisciplinary collaboration between materials specialists, engineers, and dental professionals will be important for the successful implementation of these innovations in standard clinical settings.

## Figures and Tables

**Table 1:** Historical Eras of Cementation.

Era	Philosophy	Materials	Primary Retention Mechanism	Limitations
Old Luting Era (1900s–1970s) [2,3,6]	Mechanical— Cement as a passive space-filler	Zinc phosphate, Zinc Polycarboxylate, Glass ionomer cements	Macro-mechanical (preparation geometry: taper, height, surface area).	No adhesion, high solubility, low strength, marginal degradation, required aggressive tooth preparation
Transitory Resin Cement Era (1980s–2000s) [7,8,9]	Micromechanical—First true bonding	Early Bis-GMA resin cements, Dual-cure cements, Early etch-and-rinse adhesives.	Micromechanical via resin tag formation in etched enamel; primitive hybrid layer in dentin.	Technique sensitivity, polymerization challenges under thick ceramics, hydrolytic degradation of hydrophilic adhesives
Modern Adhesive Era (2000s–Present) [4,5,10,11]	Biomimetic & Chemical— Durable, integrated interface	Universal adhesives (10-MDP), Self-etch systems, Bioactive cements	Combined micromechanical + stable chemical bonding (e.g., MDP-Ca/MDP-Zr salts)	Complexity of dentin bonding, zirconia adhesion still technique-sensitive, long-term degradation of some interfaces

**Table 2:** Historical Evolution of Luting Cements.

Cement Type	Year Introduced	Clinical Significance	Advantages	Key Limitations
Zinc Phosphate	Late 1800s	Mainstay for decades; longest clinical history	Acceptable clinical performance; low cost	No adhesion to tooth structure; high solubility; low tensile strength; requires aggressive preparation designs
Polycarboxylate	1960s	First chemically adhesive cement	Chemical bonding via calcium ion chelation	High viscosity; difficult handling; limited clinical adoption
Glass Ionomer Cement (GIC)	1970s	Milestone in adhesive dentistry	Chemical bonding to tooth; cumulative release (23.67 ppm); favorable CTE (10–14 ppm/°C) close to tooth structure	Moisture sensitivity; low fracture toughness (0.5–1.5 MPa·m^1^/^2^); solubility; limited to low-stress areas
Resin-Modified GIC (RMGIC)	1990s	Addressed GIC moisture sensitivity and initial strength	Improved mechanical properties; reduced moisture sensitivity; fluoride release	Inferior adhesive performance vs. resin cements; higher failure rates

**Table 3:** Comparative Physicochemical Properties of Luting Agents.

Property	Conventional GIC	RMGIC	Self-Adhesive Resin Cement	Adhesive Resin Cement
Flexural Strength (MPa) [16,20,43,44]	20–50	50–80	80–120	100–150
Elastic Modulus (GPa) [43,44,45]	2–6	4–8	5–10	8–15
Degree of Conversion (%) [21,22,46,47,48,49]	N/A (acid–base reaction only)	40–60	55–75	65–85
Water Sorption (µg/mm^3^) [50,51]	50–150	40–170	20–40	15–35
Solubility (µg/mm^3^) [51,52]	5–20	2–8	0.5–2.0	0.1–1.0
Cumulative Release (ppm) [53,54,55]	23.67	20.37	Not specified	Not specified
Bond Strength to Dentin (MPa) [24,25,38,56,57,58,59]	2–5	5–10	10–20	20–40
Bond Strength to Zirconia (MPa) [27,60,61]	0–2	1–3	5–15	15–30

**Table 4:** Recommended Adhesive Protocol for Glass-Ceramics.

Step	Procedure	Principal Support
Surface Pretreatment [60,86,87]	1. Etch with 4–9% hydrofluoric acid (HF) for 20–60 s. 2. Rinse thoroughly. 3. Apply silane coupling agent.	HF creates micro-retentive surface with etch depth of 10–50 µm and increases surface area by 10–20. Silane forms siloxane bonds (Si–O–Si) with ceramic and copolymerizes with resin matrix, yielding bond strengths of 25–40 MPa.
Tooth Management [26]	Selective enamel etching + immediate dentin sealing (IDS) as needed data	Maximizes enamel bond and reduces post-operative sensitivity.
Luting Agent [8,88]	Light-cure cement for thin veneers (<1.5 mm). Dual-cure cement for thicker crowns/onlays.	Light-cure offers better color stability (ΔE < 1.5 after aging). Dual-cure ensures polymerization under thick ceramics, with DC of 60–75%.
Clinical Outcome [23,75,89]	Excellent survival rates (95–98% at 5–10 years), reinforced structure.	-

**Table 5:** Recommended Adhesive Protocol for Zirconia (Polycrystalline).

Step	Procedure	Principal Support
Surface Pretreatment [5,102]	1. Airborne-particle abrasion with 50 µm Al_2_O_3_ at 0.2–0.3 MPa. 2. Clean ultrasonically. 3. Apply MDP-containing primer or universal adhesive.	Air abrasion creates micromechanical retention with surface roughness (Ra) of 0.5–1.5 µm; 10-MDP forms stable MDP-ZrO_2_ salts with bond strengths of 15–30 MPa.
Luting Agent [61,103,104]	Adhesive resin cement containing MDP. Avoid self-adhesive cements alone.	MDP-containing cements outperform non-MDP systems by 30–50% in bond strength.
Critical Note [54,105,106,107]	Saliva contamination severely inhibits bonding (50–80% reduction). Reabrade or use alkaline cleaners if contaminated.	Contamination compromises the chemical interaction between MDP and zirconia, necessitating immediate re-treatment.

**Table 6:** Recommended Adhesive Protocol for Resin-Matrix Ceramics.

Step	Procedure	Principal Support
Surface Pretreatment [5,102]	1. Airborne-particle abrasion (sandblasting) with 50 µm Al_2_O_3_. 2. Avoid HF etching (ineffective).	Sandblasting improves micromechanical retention, increasing bond strength by 40–60% compared to untreated surfaces.
Chemical Coupling [110,111,112]	Adhesive resin cement containing MDP. Avoid self-adhesive cements alone.	Bond strengths of 20–35 MPa are achievable with universal adhesive.
Luting Agent [113,114,115,116]	Saliva contamination severely inhibits bonding (50–80% reduction). Alkaline cleaners if contaminated.	Self-adhesive cements show lower bond strengths (10–20 MPa) without pretreatment.

**Table 7:** Recommended Adhesive Protocol for Indirect Composites.

Step	Procedure	Principal Support
Surface Pretreatment [109]	Airborne-particle abrasion with 50 µm Al_2_O_3_.	Increases bond strength by 50–100% compared to untreated surfaces.
Chemical Coupling [117]	Silane or universal adhesive.	Improves compatibility with resin cement; silane provides chemical coupling to glass filler particles.
Luting Agent [35,118]	Preheated composite (superior mechanical properties) or conventional resin cement.	Preheated composites offer high filler content (70–85 wt%) and strength (120–180 MPa). However, monitor for increased biodegradation (bacterial adhesion 2–3 or higher).

## Data Availability

No new data were created or analyzed in this study. Data sharing is not applicable to this article.

## Supplementary Materials

The following supporting information can be downloaded at https://www.mdpi.com/article/10.3390/jfb17070329/s1. Supplementary File S1: Search strategy.

## Abbreviations

The following abbreviations are used in this manuscript:

IDS	Immediate dentin sealing
DC	Degree of conversion
HEMA	Hydroxyethyl methacrylate
GICs	Glass ionomer cements
RMGICs	Resin-modified glass ionomer cements
MMPs	Matrix metalloproteinases
HF	Hydrofluoric acid
Al_2_O_3_	Aluminum oxide
PICN	Polymer-infiltrated ceramic network
TEGDMA	Triethylene glycol dimethacrylate

## References

[1] Özcan M., Garcia L.D.F.R., Volpato C.A.M. (2021). Bioactive Materials for Direct and Indirect Restorations: Concepts and Applications. Front. Dent. Med..

[2] Heboyan A., Vardanyan A., Karobari M.I., Marya A., Avagyan T., Tebyaniyan H., Mustafa M., Rokaya D., Avetisyan A. (2023). Dental Luting Cements: An Updated Comprehensive Review. Molecules.

[3] Manso A.P., Carvalho R.M. (2017). Dental Cements for Luting and Bonding Restorations. Dent. Clin. N. Am..

[4] Perdigão J. (2020). Current Perspectives on Dental Adhesion: (1) Dentin Adhesion–Not There Yet. Jpn. Dent. Sci. Rev..

[5] Nagaoka N., Yoshihara K., Feitosa V.P., Tamada Y., Irie M., Yoshida Y., Van Meerbeek B., Hayakawa S. (2017). Chemical Interaction Mechanism of 10-MDP with Zirconia. Sci. Rep..

[6] Gunwal M., Gohil C., Bambawale A., Parakh S., Parakh A.B., Madanala N. (2023). Clinical Response Evaluation of Two Different Luting Cements Utilized for Cementing Metal Class II Inlays: An In Vivo Study. World J. Dent..

[7] Abad-Coronel C., Naranjo B., Valdiviezo P. (2019). Adhesive Systems Used in Indirect Restorations Cementation: Review of the Literature. Dent. J..

[8] Hardy C.M.F., Bebelman S., Leloup G., Hadis M.A., Palin W.M., Leprince J.G. (2018). Investigating the Limits of Resin-Based Luting Composite Photopolymerization through Various Thicknesses of Indirect Restorative Materials. Dent. Mater..

[9] Sofan E., Sofan A., Palaia G., Tenore G., Romeo U., Migliau G. (2017). Sofan Classification Review of Dental Adhesive Systems: From the IV Generation to the Universal Type. Ann. Stomatol..

[10] Van Meerbeek B. (2020). From Buonocore’s Pioneering Acid-Etch Technique to Self-Adhering Restoratives. A Status Perspective of Rapidly Advancing Dental Adhesive Technology. J. Adhes. Dent..

[11] Carrilho E., Cardoso M., Marques Ferreira M., Marto C.M., Paula A., Coelho A.S. (2019). 10-MDP Based Dental Adhesives: Adhesive Interface Characterization and Adhesive Stability—A Systematic Review. Materials.

[12] Tavangar M.S., Jafarpur D., Bagheri R. (2017). Evaluation of Compressive Strength and Sorption/Solubility of Four Luting Cements. J. Dent. Biomater..

[13] Saleem S.S. (2024). Comparative Evaluation of Antimicrobial Activities of Different Types of Luting Cements. Erbil Dent. J..

[14] Turkistani A., Islam S., Shimada Y., Tagami J., Sadr A. (2018). Dental Cements: Bioactivity, Bond Strength and Demineralization Progression around Restorations. Am. J. Dent..

[15] Amina, Rajput G., Ahmed S., Chaturvedi S., Addas M.K., Bhagat T.V., Gurumurthy V., Alqahtani S.M., Alobaid M.A., Alsubaiy E.F. (2022). Comparison of Microleakage in Nanocomposite and Amalgam as a Crown Foundation Material Luted with Different Luting Cements under CAD-CAM Milled Metal Crowns: An In Vitro Microscopic Study. Polymers.

[16] Saran R., Upadhya N.P., Ginjupalli K., Amalan A., Rao B., Kumar S. (2020). Effect on Physical and Mechanical Properties of Conventional Glass Ionomer Luting Cements by Incorporation of All-Ceramic Additives: An In Vitro Study. Int. J. Dent..

[17] Kheur M., Kantharia N., Iakha T., Kheur S., Husain N.A.-H., Özcan M. (2020). Evaluation of Mechanical and Adhesion Properties of Glass Ionomer Cement Incorporating Nano-Sized Hydroxyapatite Particles. Odontology.

[18] Srinivasan S.R., Mathew M.G., Jayaraman J. (2023). Comparison of Three Luting Cements for Prefabricated Zirconia Crowns in Primary Molar Teeth: A 36-Month Randomized Clinical Trial. Pediatr. Dent..

[19] Varghese E., Samson R.S., Albaker S.A., Thomas A.A., Alqarni A.S., Dhanya K.B. (2023). Evaluation of Microleakage of Stainless Steel Crowns and Pedo Jacket Crowns after Cementation with Different Luting Cements. J. Pharm. Bioallied Sci..

[20] Maletin A., Knežević M.J., Koprivica D.Đ., Veljović T., Puškar T., Milekić B., Ristić I. (2023). Dental Resin-Based Luting Materials—Review. Polymers.

[21] Schneider L.F.J., Ribeiro R.B., Liberato W.F., Salgado V.E., Moraes R.R., Cavalcante L.M. (2020). Curing Potential and Color Stability of Different Resin-Based Luting Materials. Dent. Mater..

[22] Dimitriadi M., Petropoulou A., Zinelis S., Eliades G. (2024). Degree of Conversion of Dual-Cured Composite Luting Agents: The Effect of Transition Metal-Based Touch-Cure Activators. J. Dent..

[23] Yurdagüven G.Y., Çiftçioğlu E., Kazokoğlu F.Ş., Kayahan M.B. (2024). 5-Year Clinical Performance of Ceramic Onlay and Overlay Restorations Luted with Light-Cured Composite Resin. J. Dent..

[24] Cuevas-Suárez C.E., De Oliveira Da Rosa W.L., Vitti R.P., Da Silva A.F., Piva E. (2020). Bonding Strength of Universal Adhesives to Indirect Substrates: A Meta-Analysis of in Vitro Studies. J. Prosthodont..

[25] Watanabe S., Takamizawa T., Hayashi K., Aoki R., Barkmeier W., Latta M., Watanabe H., Miyazaki M. (2024). Comparing Various Resin Luting Cement Systems in Different Etching Modes Through Bond Durability and Morphological Features. Oper. Dent..

[26] Maravic T., Mazzitelli C., Mayer-Santos E., Mancuso E., Gracis S., Breschi L., Fuzzi M. (2025). Current Trends for Cementation in Prosthodontics: Part 1—The Substrate. Polymers.

[27] Krummel A., Garling A., Sasse M., Kern M. (2019). Influence of Bonding Surface and Bonding Methods on the Fracture Resistance and Survival Rate of Full-Coverage Occlusal Veneers Made from Lithium Disilicate Ceramic after Cyclic Loading. Dent. Mater..

[28] Wiegand A., Lechte C., Kanzow P. (2021). Adhesion to Eroded Enamel and Dentin: Systematic Review and Meta-Analysis. Dent. Mater..

[29] Alghazzawi T.F. (2024). Clinical Survival Rate and Laboratory Failure of Dental Veneers: A Narrative Literature Review. J. Funct. Biomater..

[30] Popescu M., Malița M., Vorovenci A., Ștețiu A.A., Perieanu V.Ș., Costea R.C., David M., Costea R.M., Ștețiu M.A., Drăguș A.C. (2025). Wet vs. Dry Dentin Bonding: A Systematic Review and Meta-Analysis of Adhesive Performance and Hybrid Layer Integrity. Oral.

[31] Costa M.J.F., Souza B.B.T.L.C.D., Sales-Junior R.A.D., Pomacóndor-Hernández C., Borges B.C.D. (2024). Does the Application of Anti-Erosive Substances on Eroded Dentin Affect Bond Strength? A Systematic Review. Odovtos-Int. J. Dent. Sci..

[32] Mokeem L.S., Garcia I.M., Melo M.A. (2023). Degradation and Failure Phenomena at the Dentin Bonding Interface. Biomedicines.

[33] Nikaido T., Tagami J., Yatani H., Ohkubo C., Nihei T., Koizumi H., Maseki T., Nishiyama Y., Takigawa T., Tsubota Y. (2018). Concept and Clinical Application of the Resin-Coating Technique for Indirect Restorations. Dent. Mater. J..

[34] Agrawal A., Nehal R., Gala K., Sachdev S.S. (2025). Immediate Dentin Sealing: Advancing Bonding Efficacy and Clinical Success. Cureus.

[35] Samartzi T.-K., Papalexopoulos D., Sarafianou A., Kourtis S. (2021). Immediate Dentin Sealing: A Literature Review. Clin. Cosmet. Investig. Dent..

[36] Varadan P., Balaji L., Manaswini D.Y., Rajan R.M. (2023). Reinforced Immediate Dentin Sealing vs. Conventional Immediate Dentin Sealing on Adhesive Behavior of Indirect Restorations: A Systematic Review. J. Contemp. Dent. Pract..

[37] Wang J., Song W., Zhu L., Wei X. (2019). A Comparative Study of the Microtensile Bond Strength and Microstructural Differences between Sclerotic and Normal Dentine after Surface Pretreatment. BMC Oral Health.

[38] Mazzitelli C., Maravic T., Josic U., Mancuso E., Generali L., Checchi V., Breschi L., Mazzoni A. (2023). Effect of Adhesive Strategy on Resin Cement Bonding to Dentin. J. Esthet. Restor. Dent..

[39] Fallahzadeh F., Safarzadeh-Khosroshahi S., Atai M. (2017). Dentin Bonding Agent with Improved Bond Strength to Dentin through Incorporation of Sepiolite Nanoparticles. J. Clin. Exp. Dent..

[40] Coelho A., Vilhena L., Antunes M., Amaro I., Paula A., Marto C.M., Saraiva J., Ferreira M.M., Carrilho E., Ramalho A. (2022). Effect of Different Cavity Disinfectants on Adhesion to Dentin of Permanent Teeth. J. Funct. Biomater..

[41] Abdullah Alsadon O. (2022). Adhesion Concepts and Techniques for Laboratory-Processed Indirect Dental Restorations. Saudi Dent. J..

[42] Barbon F.J., Isolan C.P., Soares L.D., Bona A.D., De Oliveira Da Rosa W.L., Boscato N. (2022). A Systematic Review and Meta-Analysis on Using Preheated Resin Composites as Luting Agents for Indirect Restorations. Clin. Oral Investig..

[43] Ilie N. (2021). Microstructural Dependence of Mechanical Properties and Their Relationship in Modern Resin-Based Composite Materials. J. Dent..

[44] Skapska A., Komorek Z., Cierech M., Mierzwinska-Nastalska E. (2022). Comparison of Mechanical Properties of a Self-Adhesive Composite Cement and a Heated Composite Material. Polymers.

[45] Assaf J., Hardan L., Kassis C., Bourgi R., Devoto W., Amm E., Moussa C., Sawicki J., Lukomska-Szymanska M. (2021). Influence of Resin Cement Thickness and Elastic Modulus on the Stress Distribution of Zirconium Dioxide Inlay-Bridge: 3D Finite Element Analysis. Polymers.

[46] Hajjaj M.S., Alhowirini L.F., Alghamdi R.S., Merdad Y.M., Filemban H.K., Bawazir M., Alothman K.A., Turkestani N.A., Alzahrani S.J. (2025). Effects of Preheating on the Mechanical Properties of Dental Composites. Crystals.

[47] Kelch M., Stawarczyk B., Mayinger F. (2022). Time-Dependent Degree of Conversion, Martens Parameters, and Flexural Strength of Different Dual-Polymerizing Resin Composite Luting Materials. Clin. Oral Investig..

[48] Michailidou S., Dionysopoulos D., Papadopoulos C., Naka O., Andriotis E., Fatouros D., Tolidis K. (2023). Effect of a Diode Laser (445 nm) on Polymerization Efficiency of a Preheated Resin Composite Used for Luting of Indirect Composite Restorations. Oper. Dent..

[49] Jalalian B., Golkar P., Paktinat A., Ahmadi E., Panahande S.A., Ranjbar Omrani L. (2020). Degree of Conversion of Resin-Modified Glass Ionomer Cement Containing Hydroxyapatite Nanoparticles. Front. Dent..

[50] Ilie N. (2021). Comparative Analysis of Static and Viscoelastic Mechanical Behavior of Different Luting Material Categories after Aging. Materials.

[51] Kim D.Y., Aryan N., Lawson N.C., Cheon K. (2024). Comparison of Luting Cement Solubility: A Narrative Review. Dent. J..

[52] Thomas M., Mustafa M., Karkera R., Raj A.N., Isaac L., Reddy R.N. (2016). Comparison of the Solubility of Conventional Luting Cements with That of the Polyacid Modified Composite Luting Cement and Resin-Modified Glass Ionomer Cement. J. Contemp. Dent. Pract..

[53] Singh H., Rashmi S., Pai S., Kini S. (2020). Comparative Evaluation of Fluoride Release from Two Different Glass Ionomer Cement and a Novel Alkasite Restorative Material-An in Vitro Study. Pesqui. Bras. Odontopediatria Clín. Integr..

[54] Basso G.R., Della Bona Á., Gobbi D.L., Cecchetti D. (2011). Fluoride Release from Restorative Materials. Braz. Dent. J..

[55] Pellizzari V., Michels A., Luiz S., De Souza E., Tabchoury C., Rached R. (2017). Fluoride Ion Release of Self-Adhesive Resin Cements and Their Potential to Inhibit In Situ Enamel and Dentin Demineralization. Oper. Dent..

[56] Muto R., Takamizawa T., Shiratsuchi K., Kasahara Y., Suda S., Watanabe H., Latta M.A., Miyazaki M. (2024). Influence of Luting Strategies on Dentin Bond Performance of Self-Adhesive Resin Luting Cement in Combination with a Universal Adhesive. Clin. Oral Investig..

[57] Goulart M., Borges Veleda B., Damin D., Bovi Ambrosano G.M., Coelho de Souza F.H., Erhardt M.C.G. (2018). Preheated Composite Resin Used as a Luting Agent for Indirect Restorations: Effects on Bond Strength and Resin-Dentin Interfaces. Int. J. Esthet. Dent..

[58] Topdağı B. (2025). Optimization of Bond Strength Between Heat-Polymerized PMMA and Contemporary CAD/CAM Framework Materials: A Comparative In Vitro Study. Polymers.

[59] Ashhar M., Bansal R., Bansal M., Singh D., Soni K., Samra R.K., Gupta S. (2025). Shear Bond Strength of Different Fifth-Generation Dentin Bonding Agents: An In Vitro Comparative Analysis. Cureus.

[60] Abedi F., Siahvoshi S., Babaei M., Nourmohammadi S. (2025). Comparative Analysis of Shear Bond Strength of Four Adhesive Systems on Primary Dentin. Clin. Exp. Dent. Res..

[61] Comino-Garayoa R., Peláez J., Tobar C., Rodríguez V., Suárez M.J. (2021). Adhesion to Zirconia: A Systematic Review of Surface Pretreatments and Resin Cements. Materials.

[62] Şenol M., Gürbüz A., Oyar P. (2025). Bond Strength of Conventional Resin-Based Adhesive Cement and Self-Adhesive Resin Cement to CAD-CAM Restorative Materials. BMC Oral Health.

[63] David-Pérez M., Ramírez-Suárez J.P., Latorre-Correa F., Agudelo-Suárez A.A. (2022). Degree of Conversion of Resin-Cements (Light-Cured/Dual-Cured) under Different Thicknesses of Vitreous Ceramics: Systematic Review. J. Prosthodont. Res..

[64] Da Silva F.A.S., Paschoini V.L., Cortez T.V., Corona S.A.M., Souza-Gabriel A.E. (2024). Physicochemical and Mechanical Properties of Preheated Composite Resins for Luting Ceramic Laminates. Odontology.

[65] Petropoulou A., Dimitriadi M., Zinelis S., Papathanasiou I., Eliades G. (2025). Conversion and Tack-Curing of Light-Cured Veneer Luting Agents. J. Funct. Biomater..

[66] Roesner A.J., Schmohl L., Hahnel S., Fuchs F., König A., Rauch A. (2022). Acid Resistance of Self-Adhesive Resin Luting Cements–Changes in Surface Texture Parameters and Microhardness. Dent. Mater..

[67] Desai K., Karthickraj S.M. (2024). Comparative Evaluation of Bond Strengths Between Dual Cure Resin Cement and Light Cure Resin Cement in Root Surface Indirect Restorations: An In Vitro Analysis Study. Cureus.

[68] Meharry M.R., Schwartz J., Montalvo A., Mueller D., Mitchell J.C. (2020). Comparison of 2 Self-Adhesive Resin Cements with or without a Self-Etching Primer. Gen. Dent..

[69] Barbon F.J., Moraes R.R., Isolan C.P., Spazzin A.O., Boscato N. (2019). Influence of Inorganic Filler Content of Resin Luting Agents and Use of Adhesive on the Performance of Bonded Ceramic. J. Prosthet. Dent..

[70] Garner J.R., Wajdowicz M.N., DuVall N.B., Roberts H.W. (2017). Selected Physical Properties of New Resin-Modified Glass Ionomer Luting Cements. J. Prosthet. Dent..

[71] Sriamporn T., Thamrongananskul N., Klaisiri A. (2022). The Effectiveness of Various Functional Monomers in Self-Adhesive Resin Cements on Prosthetic Materials. J. Int. Soc. Prev. Community Dent..

[72] Bharali K., Das M., Jalan S., Paul R., Deka A. (2017). To Compare and Evaluate the Sorption and Solubility of Four Luting Cements after Immersion in Artificial Saliva of Different pH Values. J. Pharm. Bioallied Sci..

[73] da Silva J.C., Rogério Vieira R., Rege I.C.C., Cruz C.A.d.S., Vaz L.G., Estrela C., Castro F.L.A. (2015). de Pre-Heating Mitigates Composite Degradation. J. Appl. Oral Sci..

[74] Penteado M.M., Tribst J.P.M., Jurema A.L.B., Saavedra G.S.F.A., Borges A.L.S. (2019). Influence of Resin Cement Rigidity on the Stress Distribution of Resin-Bonded Fixed Partial Dentures. Comput. Methods Biomech. Biomed. Eng..

[75] Hajaj T., Lile I.E., Negru R.M., Niculescu S.T., Stuparu S., Rominu M., Sinescu C., Albu P., Titihazan F., Veja I. (2025). Adhesive Performance of Zirconia and Lithium Disilicate Maryland Cantilever Restorations on Prepared and Non-Prepared Abutment Teeth: An In Vitro Comparative Study. Biomimetics.

[76] Chiapinotto G.F., Da Rosa L.S., Scotti N., Kleverlaan C.J., Valandro L.F., Pereira G.K.R. (2022). Does Adhesive Luting Promote Improved Fatigue Performance of Lithium Disilicate Simplified Crowns?. J. Mech. Behav. Biomed. Mater..

[77] Fraga S., Jager N., Campos F., Valandro L.F., Kleverlaan C.J. (2018). Does Luting Strategy Affect the Fatigue Behavior of Bonded Y-TZP Ceramic?. J. Adhes. Dent..

[78] Sundfeld D., Palialol A.R.M., Fugolin A.P.P., Ambrosano G.M.B., Correr-Sobrinho L., Martins L.R.M., Pfeifer C.S. (2018). The Effect of Hydrofluoric Acid and Resin Cement Formulation on the Bond Strength to Lithium Disilicate Ceramic. Braz. Oral Res..

[79] Alkhurays M. (2019). Influence of Different Luting Cements on the Shear Bond Strength of Pretreated Lithium Disilicate Materials. J. Contemp. Dent. Pract..

[80] Mourouzis P., Koulaouzidou E., Palaghias G., Helvatjoglu-Antoniades M. (2018). Color Match of Luting Composites and Try-in Pastes: The Impact on the Final Color of CAD/CAM Lithium Disilicate Restorations. Int. J. Esthet. Dent..

[81] Meister J., Kaschuba N., Romer M., Bourauel C. (2023). Influence of Cementation on the Aesthetical Appearance of Full-Ceramic Restorations. Materials.

[82] Caminha L.I., Dors M., Isolan C.P., Barbon F.J., Boscato N. (2022). Effect of Preheated Composite Resin Luting Agents on the Color of Ceramic Laminates. Gen. Dent..

[83] Lima L.C., Aldarvis J., Amaral F.L., Vieira-Junior W.F., Turssi C.P., Basting R.T., Lima A., Barros L.S., França F.M. (2024). Microhardness, Diametral Tensile Strength and Color Stability of Heated Resin Composites Used for Luting Ceramic Veneers. Am. J. Dent..

[84] Da Rosa L.S., Dapieve K.S., Dalla-Nora F., Rippe M.P., Valandro L.F., Sarkis-Onofre R., Pereira G.K.R. (2022). Does Adhesive Luting Reinforce the Mechanical Properties of Dental Ceramics Used as Restorative Materials? A Systematic Review and Meta-Analysis. J. Adhes. Dent..

[85] Spazzin A., Guarda G., Oliveira-Ogliari A., Leal F., Correr-Sobrinho L., Moraes R. (2016). Strengthening of Porcelain Provided by Resin Cements and Flowable Composites. Oper. Dent..

[86] Karaokutan I., Aykent F., Özdoğan M. (2023). Comparison of the Color Change of Porcelain Laminate Veneers Produced by Different Materials After Luting with Three Resin Cements. Oper. Dent..

[87] Awad M.M., Alhalabi F., Alotaibi N., Alzamil F., Binalrimal S., Alrahlah A., Ahmed M.H. (2022). A Systematic Review and Meta-Analysis of Bond Strength Studies Associated with Self-Etching Primer and HF Acid Etching of Dental Glass-Ceramics. Int. J. Adhes. Adhes..

[88] Prochnow C., Castillo D.C., Pilecco R.O., Dal Piva A.M.D.O., Tribst J.P.M., Valandro L.F., Moraes R.R.D., Pereira G.K.R. (2026). Influence of Different Evaluation Pastes on the Bond of Resin Cement to Lithium Disilicate Ceramic: An in Vitro Study. J. Prosthet. Dent..

[89] Mazzitelli C., Paolone G., Sabbagh J., Scotti N., Vichi A. (2023). Color Stability of Resin Cements after Water Aging. Polymers.

[90] Hawthan M., Larsson C., Chrcanovic B.R. (2024). Survival of Fixed Prosthetic Restorations on Vital and Nonvital Teeth: A Systematic Review. J. Prosthodont..

[91] Mihali S.G., Hiller A. (2025). State-of-the-Art Zirconia and Glass–Ceramic Materials in Restorative Dentistry: Properties, Clinical Applications, Challenges, and Future Perspectives. Appl. Sci..

[92] D’Alessandro C., Josic U., Mazzitelli C., Maravic T., Graham L., Barausse C., Mazzoni A., Breschi L., Blatz M.B. (2024). Is Zirconia Surface Etching a Viable Alternative to Airborne Particle Abrasion? A Systematic Review and Meta-Analysis of in Vitro Studies. J. Dent..

[93] Vivek V.J., Venugopal P., Divakar N., Bharath S., Sarin K., Mohammed N. (2022). Comparison of Zirconia to Dentin Bonding Using Resin-Based Luting Cements and Resin-Modified Glass-Ionomer Cement: In Vitro. J. Pharm. Bioallied Sci..

[94] Sulaiman T.A., Suliman A.A., Abdulmajeed A.A., Zhang Y. (2024). Zirconia Restoration Types, Properties, Tooth Preparation Design, and Bonding. A Narrative Review. J. Esthet. Restor. Dent..

[95] Tayal A., Niyogi A., Adhikari H., Adhya P., Ghosh A. (2021). Comparative Evaluation of Effect of One Coat 7 Universal and Tetric N-Bond Universal Adhesives on Shear Bond Strength at Resin–Zirconia Interface: An in Vitro Study. J. Conserv. Dent..

[96] Liu X., Jiang X., Xu T., Zhao Q., Zhu S. (2020). Investigating the Shear Bond Strength of Five Resin-Based Luting Agents to Zirconia Ceramics. J. Oral Sci..

[97] Ansari S., Jahedmanesh N., Cascione D., Zafarnia P., Shah K.C., Wu B.M., Moshaverinia A. (2018). Effects of an Etching Solution on the Adhesive Properties and Surface Microhardness of Zirconia Dental Ceramics. J. Prosthet. Dent..

[98] Conner C., Andretti F., Hernandez A.I., Rojas-Rueda S., Azpiazu-Flores F.X., Morrow B.R., Garcia-Godoy F., Jurado C.A., Alshabib A. (2025). Surface Evaluation of a Novel Acid-Etching Solution for Zirconia and Lithium Disilicate. Materials.

[99] Vishnu G., Jeevanandan G. (2024). Evaluation of Microleakage Using Different Luting Cements in Kedo Zirconia Crowns: An In Vitro Assessment. Cureus.

[100] Sahin I., Karayilmaz H., Çiftçi Z.Z., Kirzioglu Z. (2018). Fracture Resistance of Prefabricated Primary Zirconium Crowns Cemented with Different Luting Cements. Pediatr. Dent..

[101] Kitayama S., Nikaido T., Ikeda M., Alireza S., Miura H., Tagami J. (2010). Internal Coating of Zirconia Restoration with Silica-Based Ceramic Improves Bonding of Resin Cement to Dental Zirconia Ceramic. Bio-Med. Mater. Eng..

[102] Abhishek G., Vishwanath S.K., Nair A., Prakash N., Chakrabarty A., Malalur A.K. (2022). Comparative Evaluation of Bond Strength of Resin Cements with and without 10-Methacryloyloxydecyl Dihydrogen Phosphate (Mdp) to Zirconia and Effect of Thermocycling on Bond Strength-An in Vitro Study. J. Clin. Exp. Dent..

[103] Yang B., Barloi A., Kern M. (2010). Influence of Air-Abrasion on Zirconia Ceramic Bonding Using an Adhesive Composite Resin. Dent. Mater..

[104] Hajjaj M.S., Barboud H.M., Almashabi H.K., Alzahrani S.J., Haimed T.S.A., Alnoury A.S., Sulaiman T.A. (2023). Evaluation of Different Priming Agents with Conventional and Bioactive Self-Adhesive Resin Cements on Shear Bond Strength to Zirconia. Appl. Sci..

[105] Tyor S., Al-Zordk W., Sakrana A.A. (2023). Fracture Resistance of Monolithic Translucent Zirconia Crown Bonded with Different Self-Adhesive Resin Cement: Influence of MDP-Containing Zirconia Primer after Aging. BMC Oral Health.

[106] Krifka S., Preis V., Rosentritt M. (2017). Effect of Decontamination and Cleaning on the Shear Bond Strength of High Translucency Zirconia. Dent. J..

[107] Feitosa S., Patel D., Borges A., Alshehri E., Bottino M., Özcan M., Valandro L., Bottino M. (2015). Effect of Cleansing Methods on Saliva-Contaminated Zirconia—An Evaluation of Resin Bond Durability. Oper. Dent..

[108] Alsultani R.Z., Gholam M.K. (2025). Effectiveness of Various Cleaning Protocols in Enhancing Resin–Zirconia Bond Strength After Saliva Contamination. Prosthesis.

[109] Mine A., Kabetani T., Kawaguchi-Uemura A., Higashi M., Tajiri Y., Hagino R., Imai D., Yumitate M., Ban S., Matsumoto M. (2019). Effectiveness of Current Adhesive Systems When Bonding to CAD/CAM Indirect Resin Materials: A Review of 32 Publications. Jpn. Dent. Sci. Rev..

[110] Dikici B., Türkeş Başaran E., Can E. (2025). Does the Type of Resin Luting Material Affect the Bonding of CAD/CAM Materials to Dentin?. Dent. J..

[111] Lamberti Miotti L., Cargnelutti Follak A., De Souza Gonçalves L., Aldrighi Münchow E., Henrique Susin A. (2024). Bond Strength to Dentin of a Polymer-Infiltrate Ceramic-Network Material Cemented with Dual Resin Cements Submitted to Different Adhesive Strategies. Int. J. Adhes. Adhes..

[112] Awad M.M., Alhalabi F., Alshehri A., Salem M.A., Robaian A., Alghannam S., Alayad A.S., Almutairi B., Alrahlah A. (2023). Silane-Containing Universal Adhesives Influence Resin-Ceramic Microtensile Bond Strength. Coatings.

[113] Rohr N., Flury A., Fischer J. (2017). Efficacy of a Universal Adhesive in the Bond Strength of Composite Cements to Polymer-Infiltrated Ceramic. J. Adhes. Dent..

[114] Calheiros-Lobo M.J., Vieira T., Carbas R., Da Silva L.F.M., Pinho T. (2023). Effectiveness of Self-Adhesive Resin Luting Cement in CAD-CAM Blocks—A Systematic Review and Meta-Analysis. Materials.

[115] Berkman M., Tuncer S., Tekçe N., Karabay F., Demirci M. (2020). Microtensile Bond Strength Between Self-Adhesive Resin Cements and Resin Based Ceramic CAD/CAM Block. Odovtos-Int. J. Dent. Sci..

[116] Bayas Salinas A., Reascos Flores A., Cascante-Calderón M.G., Villacís Altamirano I.M. (2025). Effect of Thermocycling and Surface Treatments on the Bond Strength of a Hybrid PICN Ceramic. Acta Odontol. Latinoam..

[117] Matinlinna J.P., Lung C.Y.K., Tsoi J.K.H. (2018). Silane Adhesion Mechanism in Dental Applications and Surface Treatments: A Review. Dent. Mater..

[118] Bezerra A., Gonçalves G., Alves L., Stamfor T., De Brito O., Monteiro G. (2024). Bacterial Adhesion and In Situ Biodegradation of Preheated Resin Composite Used as a Luting Agent for Indirect Restorations. Oper. Dent..

[119] Alsunbul H., Khan A.A., Alqahtani Y.M., Hassan S.A.B., Asiri W., Saadaldin S., Alharthi R., Aldegheishem A. (2023). Using Functionalized Micron-Sized Glass Fibres for the Synergistic Effect of Glass Ionomer on Luting Material. J. Funct. Biomater..

[120] Pandey A. (2024). Recent Advances in Bioactive Dental Materials: A Paradigm Shift in Restorative Dentistry. Dental.

[121] Zailai A., Mubarki O., Alobaidan A.N., Alenazi S.A., Humedi A.A., Alshahrani K.M., Alfattah K.M., Baobied M.S., Alenizi T., Eishan A.A. (2026). Clinical Efficacy of Bioactive and Smart Restorative Materials in Preventing Secondary Caries: A Systematic Review and Meta-Analysis. Cureus.

[122] Zhang K., Zhang N., Weir M.D., Reynolds M.A., Bai Y., Xu H.H.K. (2017). Bioactive Dental Composites and Bonding Agents Having Remineralizing and Antibacterial Characteristics. Dent. Clin. N. Am..

[123] Wu J., Zhang Q., Weir M.D., Oates T.W., Zhou C., Chang X., Xu H.H.K. (2017). Novel Self-Healing Dental Luting Cements with Microcapsules for Indirect Restorations. J. Dent..

[124] Wang X., Ding T. (2024). A Review on the Current State of Microcapsule-Based Self-Healing Dental Composites. J. Funct. Biomater..

[125] Bacchi A., Spazzin A.O., De Oliveira G.R., Pfeifer C., Cesar P.F. (2018). Resin Cements Formulated with Thio-Urethanes Can Strengthen Porcelain and Increase Bond Strength to Ceramics. J. Dent..

[126] Ishikawa K., Yamauti M., Tichy A., Ikeda M., Ueno T., Wakabayashi N., Thanatvarakorn O., Prasansuttiporn T., Klein-Junior C.A., Takahashi A. (2021). UV-Mediated Photofunctionalization of Indirect Restorative Materials Enhances Bonding to a Resin-Based Luting Agent. BioMed Res. Int..

[127] Vohra F., Altwaim M., Alshuwaier A., Al Deeb M., Alfawaz Y., Alrabiah M., Abduljabbar T. (2020). Influence of Bioactive, Resin and Glass Ionomer Luting Cements on the Fracture Loads of Dentin Bonded Ceramic Crowns: Failure Loads of Ceramic Crowns Bonded with Bioactive Cement. Pak. J. Med. Sci..

[128] Komatsu K., Suzumura T., Komatsu E., Matsuura T., Shibata R., Kusunoki Y., Choi J., Ogawa T. (2025). Reimagining Bonding Interfaces: UV Photofunctionalization, a Novel Physicochemical Approach, Unlocks Titanium and Cement Adhesive Potential. Dent. Mater..

[129] Tribst J.P.M., Etoeharnowo L., Tadros M., Feilzer A.J., Werner A., Kleverlaan C.J., Dal Piva A.M.D.O. (2023). The Influence of Pre-Heating the Restoration and Luting Agent on the Flexural Strength of Indirect Ceramic and Composite Restorations. Biomater. Investig. Dent..

[130] Falacho R.I., Marques J.A., Palma P.J., Roseiro L., Caramelo F., Ramos J.C., Guerra F., Blatz M.B. (2022). Luting Indirect Restorations with Resin Cements versus Composite Resins: Effects of Preheating and Ultrasound Energy on Film Thickness. J. Esthet. Restor. Dent..

[131] Gerdolle D., Browet S., Gresnigt M. (2024). The Multi-Luting Concept: A New Approach to Facilitate the Adhesive Luting of Multiple Indirect Restorations. Swiss Dent. J. SSO.

[132] Elkaffas A.A., Alshehri A., Alqahtani A.A., Almudahi A.F., Alanazi K.K., Alhalabi F.A., Abuelqomsan M.A., Alqahtani A.R. (2025). Fracture Resistance of Milled and 3D Printed Ultra-Thin Occlusal Veneers Made of CAD/CAM Resin-Based Ceramics Cemented by Variable Luting Approaches. BMC Oral Health.

[133] Alshabib A., Berger J., Garcia E., Jurado C.A., Cabral G., Baldotto A., Riquieri H., Alrabiah M., Floriani F. (2026). A Comprehensive Digital Workflow for Enhancing Dental Restorations in Severe Structural Wear. Bioengineering.

[134] Alghauli M.A., Alqutaibi A.Y., Wille S., Kern M. (2023). Clinical Reliability of Self-Adhesive Luting Resins Compared to Other Adhesive Procedures: A Systematic Review and Meta-Analysis. J. Dent..

[135] Maroulakos G., Thompson G.A., Kontogiorgos E.D. (2019). Effect of Cement Type on the Clinical Performance and Complications of Zirconia and Lithium Disilicate Tooth-Supported Crowns: A Systematic Review. Report of the Committee on Research in Fixed Prosthodontics of the American Academy of Fixed Prosthodontics. J. Prosthet. Dent..

[136] Ghodsi S., Arzani S., Shekarian M., Aghamohseni M. (2021). Cement Selection Criteria for Full Coverage Restorations: A Comprehensive Review of Literature. J. Clin. Exp. Dent..

[137] Taschner M., Stirnweiss A., Frankenberger R., Kramer N., Galler K.M., Maier E. (2022). Fourteen Years Clinical Evaluation of Leucite-Reinforced Ceramic Inlays Luted Using Two Different Adhesion Strategies. J. Dent..

[138] Ashraf H., El Tannir A., El Zohairy A., Kamal D. (2025). Clinical Performance of Indirect Hybrid Ceramic Onlay Restorations Cemented with Injectable Resin Composite versus Dual-Cure Resin Cement: An 18-Month Randomized Clinical Trial. BMC Oral Health.

[139] Ding J., Jin Y., Feng S., Chen H., Hou Y., Zhu S. (2022). Effect of Temporary Cements and Their Removal Methods on the Bond Strength of Indirect Restoration: A Systematic Review and Meta-Analysis. Clin. Oral Investig..

[140] Eltoukhy R.I., Elkaffas A.A., Ali A.I., Mahmoud S.H. (2021). Indirect Resin Composite Inlays Cemented with a Self-Adhesive, Self-Etch or a Conventional Resin Cement Luting Agent: A 5 Years Prospective Clinical Evaluation. J. Dent..

[141] Hardan L., Devoto W., Bourgi R., Cuevas-Suárez C.E., Lukomska-Szymanska M., Fernández-Barrera M.Á., Cornejo-Ríos E., Monteiro P., Zarow M., Jakubowicz N. (2022). Immediate Dentin Sealing for Adhesive Cementation of Indirect Restorations: A Systematic Review and Meta-Analysis. Gels.

[142] Staněk J., Riad A., Le A., Bernát M., Hammal M., Azar B. (2022). Survival of Prosthodontic Restorations Luted with Resin-Based versus Composite-Based Cements: Retrospective Cohort Study. Materials.

[143] Brunton P.A., Ratnayake J., Loch C., Veerasamy A., Cathro P., Lee R. (2019). Indirect Restorations and Fixed Prosthodontics: Materials and Techniques Used by General Dentists of New Zealand. Int. J. Dent..

[144] Mohamed S.G.A., Hussein H.G.A., Mohamed G.A., Ibrahim S.R.M. (2025). Different Zirconia Surface Treatments: Strategies to Enhance Adhesion in Zirconia-Based Dental Restorations. J. Pharm. Bioallied Sci..

[145] Alsaeed A.Y. (2022). Bonding CAD/CAM Materials with Current Adhesive Systems: An Overview. Saudi Dent. J..

[146] Şahan M.H., Peşkersoy C., Kümbüloğlu O., Türkün M. (2023). Effect of Different Adhesive Systems and Silane Application on Shear Bond Strength of Resin Cement to Indirect Restorations. J. Dent. Mater. Tech..

[147] Siqueira F., Cardenas A.M., Gutierrez M.F., Malaquias P., Hass V., Reis A., Loguercio A.D., Perdigao J. (2016). Laboratory Performance of Universal Adhesive Systems for Luting CAD/CAM Restorative Materials. J. Adhes. Dent..

[148] Conejo J. (2022). Current Adhesive Protocols for Indirect Ceramic Restorations. Compend. Contin. Educ. Dent..

[149] Habibzadeh S., Khamisi F., Mosaddad S.A., Fernandes G.V.D.O., Heboyan A. (2024). Full-Ceramic Resin-Bonded Fixed Dental Prostheses: A Systematic Review. J. Appl. Biomater. Funct. Mater..

[150] Kolanko J., Bonsor S. (2022). Does Immediate Dentine Sealing Improve Bonding Effectiveness of Glass Ceramic Restorations Compared to Delayed Dentine Sealing?. Eur. J. Prosthodont. Restor. Dent..

[151] Bragança G.F.D., Mazão J.D., Versluis A., Soares C.J. (2021). Effect of Luting Materials, Presence of Tooth Preparation, and Functional Loading on Stress Distribution on Ceramic Laminate Veneers: A Finite Element Analysis. J. Prosthet. Dent..

[152] Politano G., Van Meerbeek B., Peumans M. (2018). Nonretentive Bonded Ceramic Partial Crowns: Concept and Simplified Protocol for Long-Lasting Dental Restorations. J. Adhes. Dent..

[153] Solon-de-Mello M., Da Silva Fidalgo T.K., Dos Santos Letieri A., Masterson D., Granjeiro J.M., Monte Alto R.V., Maia L.C. (2019). Longevity of Indirect Restorations Cemented with Self-adhesive Resin Luting with and without Selective Enamel Etching. A Systematic Review and Meta-analysis. J. Esthet. Restor. Dent..

[154] Alvarenga M., Machado L., Prado A., Veloso S., Monteiro G. (2024). Self-Adhesive Resin Cement versus Conventional Cements on the Failure Rate of Indirect Single-Tooth Restorations: A Systematic Review and Meta-Analysis of Randomized Clinical Trials. J. Prosthet. Dent..

[155] Magne P., Razaghy M., Carvalho M.A., Soares L.M. (2018). Luting of Inlays, Onlays, and Overlays with Preheated Restorative Composite Resin Does Not Prevent Seating Accuracy. Int. J. Esthet. Dent..

[156] Hardy C.M.F., Landreau V., Valassis M., Mercelis B., De Munck J., Van Meerbeek B., Leprince J. (2021). Mini-iFT Confirms Superior Adhesive Luting Performance Using Light-Curing Restorative Composites. J. Adhes. Dent..

[157] Labunet A., Kui A., Vigu A., Voina-Tonea A., Burde A., Sava S. (2026). Preheated Composite for Prosthetic Cementation to Enamel and Dentin: A Scoping Review. Dent. J..

[158] Chiodera G., Monterubbianesi R., Tosco V., Papini O., Orsini G., Putignano A. (2024). Application of Bulk-Fill Composite to Simplify the Cementation of Indirect Restorations: The COMBO Technique. Dent. J..

[159] Tsujimoto A., Barkmeier W.W., Takamizawa T., Watanabe H., Johnson W.W., Latta M.A., Miyazaki M. (2018). Simulated Localized Wear of Resin Luting Cements for Universal Adhesive Systems with Different Curing Mode. J. Oral Sci..

[160] Hoffmann L., Kessler A., Kunzelmann K.-H. (2021). Three-Body Wear of Luting Composites and Influence of the ACTA Wheel Material. Dent. Mater. J..

[161] Oshika M., Kishimoto T., Horie T., Alhotan A., Irie M., Sule V.C., Barkmeier W.W., Tsujimoto A. (2024). Wear Resistance of Light-Cure Resin Luting Cements for Ceramic Veneers. J. Funct. Biomater..

[162] Tosco V., Monterubbianesi R., Orilisi G., Sabbatini S., Conti C., Özcan M., Putignano A., Orsini G. (2021). Comparison of Two Curing Protocols during Adhesive Cementation: Can the Step Luting Technique Supersede the Traditional One?. Odontology.

[163] Stegall D., Tantbirojn D., Perdigao J., Versluis A. (2017). Does Tack Curing Luting Cements Affect the Final Cure?. J. Adhes. Dent..

[164] Breschi L., Josic U., Maravic T., Mancuso E., Del Bianco F., Baldissara P., Mazzoni A., Mazzitelli C. (2023). Selective Adhesive Luting: A Novel Technique for Improving Adhesion Achieved by Universal Resin Cements. J. Esthet. Restor. Dent..

[165] Ishii R., Takamizawa T., Katsuki S., Iwase K., Shoji M., Sai K., Tsujimoto A., Miyazaki M. (2022). Immediate Bond Performance of Resin Composite Luting Systems to Saliva-contaminated Enamel and Dentin in Different Curing Modes. Eur. J. Oral Sci..

[166] Buyukates I., Garoushi S., Vallittu P.K., Uctasli S., Lassila L. (2026). Effect of Different Luting Protocols on the Bond Strength of Fiber-Reinforced CAD/CAM Blocks. Polymers.

[167] Merlo E.G., Della Bona A., Griggs J.A., Jodha K.S., Corazza P.H. (2020). Mechanical Behavior and Adhesive Potential of Glass Fiber-Reinforced Resin-Based Composites for Use as Dentin Analogues. Am. J. Dent..

[168] Alshabib A., AlDosary K., Algamaiah H. (2024). A Comprehensive Review of Resin Luting Agents: Bonding Mechanisms and Polymerisation Reactions. Saudi Dent. J..

[169] Leung G.K.-H., Wong A.W.-Y., Chu C.-H., Yu O.Y. (2022). Update on Dental Luting Materials. Dent. J..

