# Supplementary material for: Functional Adhesives for a Restorative Future
Source: J Funct Biomater. 2026 Jul 6;17(7):329. doi: 10.3390/jfb17070329 (PMC13412557; doi:10.3390/jfb17070329)
Supplement: Supplementary file 1 [file jfb-17-00329-s001.zip › jfb-4279653-supplementary.pdf]

Supplementary File S1
Keywords and Subject Headings Used During the Search

Database	Search strategy
PubMed accessed January 2015- March 2026	("Dental Cementum"[MeSH] OR "Resin Cements"[MeSH] OR "Cementation"[MeSH] OR "Luting" OR "Adhesive cementation" OR "Resin cement" OR "Self-adhesive cement" OR "Universal adhesive")<br>AND<br>("Zirconia"[MeSH] OR "Composite Resins"[MeSH] OR "Ceramics"[MeSH] OR "Lithium disilicate" OR "Leucite" OR "Zirconia" OR "Hybrid ceramic" OR "PICN" OR "Resin-matrix ceramic" OR "Indirect composite")<br>AND<br>("Dental Bonding"[MeSH] OR "Shear Strength"[MeSH] OR "Dentin-Bonding Agents"[MeSH] OR "Tooth preparation" OR "Enamel" OR "Dentin" OR "Immediate dentin sealing" OR "Bond durability" OR "Clinical outcome" OR "Survival")
Scopus accessed January 2015- March 2026	("resin cements" OR "luting" OR "adhesive cementation" OR "universal adhesive")<br>AND<br>(“ceramics” OR "zirconia" OR "composite resins " OR "lithium disilicate" OR "hybrid ceramic" OR "resin-matrix ceramic" OR "indirect composite")<br>AND<br>("tooth preparation” OR "enamel " OR "dentine " OR "dental bonding " OR " immediate dentin sealing " OR " dentin-bonding agents" OR " bond durability " OR " clinical outcome ")<br>AND<br>PUBYEAR > 2015 AND PUBYEAR < 2026
Web Of Science accessed January 2015- March 2026	TS=(“ceramics” OR"zirconia" OR "composite resins " OR "lithium disilicate" OR "hybrid ceramic" OR "resin-matrix ceramic" OR "indirect composite")<br>AND<br>TS=("resin cements" OR "luting" OR "adhesive cementation" OR "universal adhesive")<br>AND<br>TS=("tooth preparation” OR "enamel " OR "dentine " OR "dental bonding " OR " immediate dentin sealing " OR " dentin-bonding agents" OR " bond durability" OR " clinical outcome ")<br>AND<br>PY=(2015-2026)
